# The Impact of Changes in Professional Autonomy and Occupational Commitment on Nurses’ Intention to Leave: A Two-Wave Longitudinal Study in Japan

**DOI:** 10.3390/ijerph17176120

**Published:** 2020-08-22

**Authors:** Yukari Hara, Kyoko Asakura, Takashi Asakura

**Affiliations:** 1 Graduate School of Medicine, Tohoku University, 2-1, Seiryo-machi, Aoba-ku, Sendai, Miyagi 980-8575, Japan; asakura@med.tohoku.ac.jp; 2 Faculty of Education, Tokyo Gakugei University, 4-1-1, Nukuikita, Koganei, Tokyo 184-8501, Japan; asakurat@u-gakugei.ac.jp

**Keywords:** professional autonomy, intention to leave, Japan, nurses, occupational commitment, turnover

## Abstract

This study aimed to investigate changes in nurses’ attitudes toward professional autonomy and occupational commitment over time, and their effect on nurses’ intentions to leave, using a two-wave longitudinal design. Anonymous, self-report questionnaires were distributed to all nurses working at 28 hospitals in western Japan on two separate occasions (*n* = 1778). Multivariate analysis using a generalized estimation equation was conducted, with the intention to leave at Time 2 as the dependent variable, and the changing secular trends in all subscales of attitudes toward professional autonomy and occupational commitment as the independent variables. Age, sex, education, and intention to leave at Time 1 were control variables. Results showed that increasing changing secular trends in control over work conditions, which is a subscale of attitudes toward professional autonomy, increased intention to leave at Time 2, while increasing changing secular trends in all subscales of occupational commitment decreased intention to leave at Time 2. Nurses with a progressive attitude toward discretion of control over work conditions may have higher intentions to leave. Therefore, increasing control over their work conditions may reduce this intention. Additionally, it is necessary to continually enhance nurses’ occupational commitment by offering professional development programs.

## 1. Introduction

The rapid aging of the global population in recent years has increased the burden of disease and the need for healthcare services. In that context, providing high-quality and safe medical care is a central issue for nurses worldwide [1]. In order to solve this problem, nurses’ professional autonomy and occupational commitment have recently been gaining attention as factors that enhance the practical ability, quality, and safety of the care services they provide [2,3,4,5,6].

Healthcare services around the world have long been experiencing a serious shortage of nurses [7]. With the goal of reducing nurse shortages, research has focused on nurses’ intention to leave the job, since it strongly predicts their turnover behavior [8]. It has been reported that their intention to leave is associated with their mental and physical health, as well as psychosocial factors. For example, nurses’ mental health is poorer than that of the general worker population, and the odds that they will leave their job increase with mental health deterioration [9]. Moreover, nurses’ intention to leave their job increases when their self-ratings for health are poor [10]. Other studies indicate that working long shifts and bad working environments significantly increase their intention to leave [11,12]. Further, job stress directly or indirectly affects intention to leave [13].

Reports on professional autonomy [14,15] and occupational commitment [16,17,18,19] as predictors of nurses’ intention to leave have increased. Professional autonomy is an integral part of the medical profession. It refers to the control of work, independence, and the ability to employ clinical decision making and clinical judgment regarding patient care within the scope of an individual’s profession [3,20]. There is some evidence that the higher nurses’ professional autonomy, the greater the safety [2] and work performance [3], and the lower the mortality rate and failure to rescue rates of the patients [5,6]. Moreover, higher levels of professional autonomy lead to better outcomes for the nurses themselves: increased job satisfaction [3,21], improved moral distress [21,22], and reduced depression and absenteeism [23]. Thus, retaining nurses with a high level of professional autonomy in healthcare services would allow the provision of high-quality and safe medical care while maintaining the nurses’ own well-being.

However, the findings regarding the effects of nurses’ professional autonomy on their intention to leave their profession are inconsistent. Reports of licensed nurses working in the United States and registered nurses working in a pediatric hospital in Brazil revealed that nurses who are planning to quit their current jobs have lower professional autonomy than those who are planning to continue working [14,15]. However, a study on registered nurses in the Philippines reported that nurses’ professional autonomy did not significantly affect turnover intentions [3]. These differences in the results regarding whether professional autonomy influences the intention to leave suggest that nurses’ professional autonomy may not have been adequately measured, and reflected the context of the country in which the study was conducted. As the range of discretion allowed to nurses varies from country to country, it is necessary to clarify how nurses’ professional autonomy influences their intention to leave in the context of their country by appropriately measuring their professional autonomy.

Previous studies have pointed out that traditional, religious, economic, political, social, and cultural factors can affect nurses’ professional autonomy, and these factors and their impact level can vary from country to country [24]. In addition, their autonomous behavior is controlled by the national government’s laws and regulations, health systems, and the degree of discretion recognized by professional associations [25]. In the United Kingdom, Australia, the United States, and New Zealand, nurses have legal authority to demand patient medication, but this is not the case in many other countries, such as Japan and Turkey [24,25]. This indicates that, compared to the countries where the discretion of nurses is recognized, discretion is limited in countries where doctors’ authority is strong because of social and cultural factors [24,26,27]. Specifically, in Japan, nurses cannot legally intervene or conduct a medical intervention based on their own judgment; they must have a physician instruct them to conduct the intervention [27]. Furthermore, in Japan, Turkey, and Iran, nurses, who are mostly women, may have their voices restricted because of patriarchal cultural factors that persist in society [24,27]. Therefore, in patriarchal clinical settings, it is almost impossible for a female nurse to act autonomously. For these reasons, it is inadequate to measure the behavioral aspects of nurses’ professional autonomy in countries where autonomous behavior is restricted.

Nurses in countries with limited professional autonomy, such as Japan, Turkey, and Iran, seem to have a conservative attitude toward autonomy because they have difficulty acting autonomously in their physician-dominated context. This attitude will prevent them from taking up opportunities to act autonomously even when opportunities are available. Therefore, a more liberal attitude toward nurses’ professional autonomy is essential. Thus, in this study, we used the Attitude Toward Professional Autonomy Scale [27], which focuses on the cognitive aspects of nurses’ professional autonomy to measure the degree of nurses’ liberal attitudes toward professional autonomy. This cognitive aspect is based on a three-element model of attitude [28]; in other words, it measures the autonomy as perceived by the nurses and expressed in their attitudes. This scale has three subscales: (1) “autonomous clinical judgment,” which represents the attitude of taking care of patients and proceeding with work based on autonomous clinical judgment; (2) “control over work conditions,” which represents the attitude of trying to decide about work style, and (3) “job-related independence,” which indicates the attitude of nurses in trying to carry out their work without being controlled by others [27]. In this manner, by appropriately evaluating the situation in countries where nurses’ professional autonomy is limited, and by measuring their attitudes toward professional autonomy, we clarify how nurses’ professional autonomy influences their intention to leave in the context of their country.

Occupational commitment (OC), on the other hand, is widely defined as the psychological link between a person and his or her occupation [29,30], and is considered a factor affecting nurses’ intentions to leave. A large body of literature demonstrates that higher levels of nurses’ OC have a good impact on patients and nurses, for example, by affecting patient safety and perceived quality of care [31]. In addition, new nurses with higher OC are more competent and have higher job satisfaction [4]. Furthermore, the higher the OC of nurses, the higher their job satisfaction and intention to stay [32]. Therefore, it is essential to retain nurses with higher OC to provide high-quality and safe medical care while ensuring the nurses’ own well-being.

Indeed, there is evidence that nurses’ OC is a strong predictor of their intention to leave [17]. OC has three components: affective, continuance, and normative [33]. If any of these components increase, the intention to leave decreases [16] and the intention to stay increases [19]. In new nurses with low intention to leave their job, the highest measured component of OC is affective, followed by normative [18]. Therefore, it is necessary to examine the impact of OC on the intention to leave of nurses of varying ages and cultures.

Moreover, several sources have shown that professional autonomy and OC may change over time in individuals and groups. Because professional autonomy and OC are related to the quality of care, provision of safe medical care, and nursing staff’s intention to leave, their reduction may lead to the departure of nurses, deterioration of care quality, and obstruction of safe medical care. In addition, the knowledge of the changes occurring over time in nurses’ professional autonomy and OC is important because it helps hospital managers, nurse educators, and nurses themselves to understand nurses’ changing attitudes toward their profession. The amount of change in variables can be quantified by two independent indices: rank-order stability and change in mean level [34]. Rank-order stability refers to the stability of the relative position of the variables in individuals over time, indexed by the correlation between the same scores measured at two time points (i.e., the test-retest correlation) [35]. Change in mean level refers to how a variable level, averaged across a sample of individuals, might change over time [34]. The only rank-order stability report for nurses’ OC was affective OC, with *r* = 0.68 (6 months) [36]. The coefficient of personality traits, which was said to be stable, was *r* = 0.70 (average of five personality trait factors for four years) [37], and the coefficient of individual values was *r* = 0.60 (average of ten values for two years) [38]. These suggest the stability of the nurses’ affective OC. However, in the ten studies that did not mention the occupation of the subject described in the OC review paper, the lowest coefficient of affective OC was *r* = 0.47 (12 months), while the highest was *r* = 0.83 (12 months) [30]. This suggests that rank-order stability may vary depending on the subject and measurement interval, indicating the need to assess the stability of the three subscales over longer spans.

Regarding the average stability of OC, the only article that referred to the three commitment dimensions showed a mean decrease of affective OC, weak mean decrease of normative OC, and no mean change for continuance OC, within a sample of student nurses across a time span of 12 months [33]. A report determining paired samples *t*-tests for affective OC in a sample of nurses indicated that there was a statistically significant difference at Time 1 and Time 2 across a span of six months [36]. These studies consistently report an average reduction in affective OC. However, they measure the beginning and end of the academic year for nursing students [33], and before and after the entry of nurses who are employed in a new workplace [36]; thus caution is required in interpreting the results.

Regarding professional autonomy, nurses with many years of experience have been reported to have higher professional autonomy [39], but since longitudinal studies have not been conducted, there is no report to our knowledge on rank-order stability or average stability of professional autonomy. Previous studies have reported OC changes in the span of six months after entering a new workplace [36] and in 12 months for nursing students [30,33]. However, it is necessary to measure changes in nurses of all ages across several years. This study reveals relatively long-span changes in both of these variables, with measurements taken approximately two years apart.

Until now, it has been pointed out that professional autonomy and OC influence the intention to leave and that they change over time. However, the relationship between professional autonomy, OC, and intention to leave has been clarified primarily by using cross-sectional studies, instead of longitudinal ones that help measure long-term changes. This study contributes to the literature by using the changes in professional autonomy and OC measured about two years apart as the independent variables and nurses’ intention to leave as the dependent variable. Furthermore, while intention to leave the job is a strong predictor of nurses’ actual turnover [8], there are individual differences that increase/decrease turnover behavior [40]. It has also been reported that there are nurses who immediately experience increases in their intention to leave, which results in the nurses quitting. On the other hand, there are nurses who have gradual general behavior changes toward staying even though they had high intentions to leave [41]. Therefore, in this study, the problem is not nurses’ continued high intention to leave, but the high intention to leave at a certain point. We challenge this problem by using Time 2 intention to leave as the dependent variable and Time 1 intention to leave as the control variable, instead of using the amount of change in intention to leave. Knowledge of the causal relationship between the long-term change of professional autonomy and OC and intention to leave will provide information to hospital managers and nurse educators about which aspects of autonomy and commitment they should consider when deciding on appropriate turnover prevention measures for nurses.

**Figure 1:**
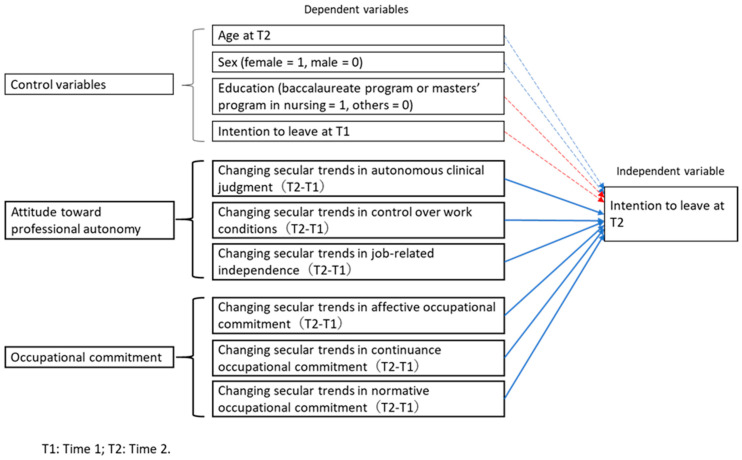
The organization of the variables in this study and their connections to each other. The solid blue arrow points to the hypothesis that the intention to leave at T2 decreases with increasing changes in secular trends in all subscales of nurses’ professional autonomy and OC (occupational commitment). The blue dotted arrows represent the results of previous studies—that older and female nurses have lower intentions to leave, and the red dotted arrows show that the higher the educational background and increase in intention to leave at T1, the higher the increase in intention to leave at T2.

Based on the above literature review, the following hypothetical model was created for the causal relationships between nurses’ professional autonomy, OC, and intention to leave (Figure 1). The model depicts two hypotheses: (1) increasing secular changes in nurses’ professional autonomy (autonomous clinical judgment, control over work condition, and job-related independence) decreases their intention to leave, and (2) increasing secular changes in nurses’ OC (affective OC, continuance OC, normative OC) decreases their intention to leave. Since age, sex, and educational background have been found to generally influence nurses’ intention to leave [8,10], they served as control variables. In the same way, it was assumed that Time 1′s intention to leave affects Time 2′s intention to leave, so it was also used as a control variable.

Thus, the main purpose of this study was to examine the effects of the changes in nurses’ professional autonomy and OC on their intention to leave. To this end, we first clarify individual and group secular changes in professional autonomy and OC. We then reveal the causal relationship between professional autonomy, OC, and intention to leave by considering the changes in these variables. In this way, by clarifying individual and group secular changes in professional autonomy and OC that have not been revealed so far by the accumulated cross-sectional studies, we provide new, useful knowledge. Specifically, as nurses’ professional autonomy and OC enhance nurses’ practical abilities and quality of care, thereby improving their on-the-job performance, this knowledge is relevant for nurses worldwide to provide high-quality and safe medical care. Furthermore, identifying how changes in professional autonomy and OC impact nurses’ intention to leave can help determine how to retain nurses with high professional autonomy and OC in the healthcare field.

## 2. Methods

This study used data from a large-scale, three-wave, longitudinal study; part of the baseline data has already been reported by Asakura et al. [16].

### 2.1. Design, Setting, and Participants

We distributed anonymous, self-report questionnaires to all nurses who were not currently on maternity, childcare, or sick leave across 28 hospitals in the same affiliated organization in western Japan (*n* = 11,171) on two separate occasions (January 2014—Time 1; October 2015—Time 2). Participants included nurses, assistant nurses, midwives, and public health nurses working at 28 hospitals. We chose the hospitals from this affiliated organization because they are advanced treatment hospitals and their standard nurse-to-patient ratio is 1:7. The number of nursing staff in those hospitals ranged from 100 to 800.

At Time 1, questionnaires and personal information sheets were distributed to participants via the nurse manager at their respective hospitals. Participants were able to return the completed questionnaire by mail or online. If the participant chose to use the ID, password, and URL of the research homepage as provided on the questionnaire to answer the electronic version, the completed survey was returned online. After an online response, we conducted a follow-up survey using participants’ e-mail addresses. If the completed questionnaire was returned by mail, we created a database from the returned personal information sheet for the follow-up survey. At Time 2, the researchers sent questionnaires by mail or email, depending on the selection of participants at Time 1. The questionnaire contained information about nurses’ demographics, intention to leave their current jobs, and their attitudes toward professional autonomy and OC.

### 2.2. Ethical Considerations

The ethics committee at the Tohoku University School of Medicine granted approval for this study (no. 2013-144) on June 24, 2013. Official permission was obtained from the hospitals where the participants had been working. Participants’ confidentiality and anonymity during the study and publication process were addressed. We informed participants about the purpose and design of the study and that participation was voluntary. Returned/submitted questionnaires were deemed an agreement to participate. This study conformed to the provisions of the Declaration of Helsinki in 1995 (as revised in Edinburgh, 2000).

### 2.3. Measures and Data Collection

#### 2.3.1. Demographic Variables

Data on individual and work-related variables were collected: sex, age, marital status, education background, years of nursing experience, employment conditions, and position.

#### 2.3.2. Attitude toward Professional Autonomy

In this study, we used a scale developed by Asakura et al. [27], which measures nurses’ attitudes toward professional autonomy. There were three subscales: autonomous clinical judgment (seven items), control over work conditions (six items), and job-related independence (five items). Responses were rated on a five-point Likert-type scale, ranging from 1 (strongly disagree) to 5 (strongly agree). Higher scores indicate a more progressive attitude toward professional autonomy. Asakura et al. [27] reported adequate internal consistency (Cronbach’s αs in this study at Time 1 = 0.85, 0.77, 0.80, and 0.87 for all the items and the three subscales, respectively) and adequate concurrent validity, which was supported by correlations with the Desire for Self-Determination Scale (*rs* = 0.14, 0.25, and 0.36 for the three subscales, respectively; *p* < 0.01).

#### 2.3.3. Occupational Commitment

To assess OC, the Japanese version of the Allen and Meyer Three-Dimensional Commitment Scale, which was translated and validated by Satoh, Asakura, Watanabe, and Shimojo [42], was used. The scale contains three subscales: affective OC (six items), continuous OC (five items), and normative OC (six items). Responses were rated on a five-point Likert-type scale, ranging from 1 (strongly disagree) to 5 (strongly agree), with higher scores indicating higher OC. Satoh et al. [42] reported adequate internal consistency (Cronbach’s α in this study at Time 1 = 0.85, 0.85, 0.80, and 0.72 for all items and the three subscales, respectively) and adequate concurrent validity, which was supported by correlations with job satisfaction (*rs* = 0.34, 0.17, 0.22 for the three subscales, respectively; *p* < 0.001).

#### 2.3.4. Intention to Leave

The Intention to Leave scale developed by Tei and Yamazaki [43] was used as the dependent variable. The scale is composed of six items on participants’ thoughts and behaviors related to resigning from their position. Responses were rated on a four-point Likert-type scale, ranging from 1 (none) to 4 (frequently), with higher scores indicating a stronger intention to leave. Tei and Yamazaki [43] reported adequate factor validity and internal consistency (Cronbach’s α in this study at Time 1 = 0.90).

### 2.4. Data Analyses

Descriptive statistics for personal and work-related attributes were calculated. Then, a factor analysis and internal consistency test of the scale (Cronbach’s α) were calculated. Next, we calculated the correlation coefficients for all independent variables and performed a paired *t*-test on professional autonomy and OC at Time 1 and Time 2. Finally, multivariate analyses using a generalized estimating equation (GEE) were conducted to verify the hypotheses. We estimated robust standard errors to account for clustering within the hospitals. Intention to leave during Time 2 was the dependent variable. In order to explore the independent variables that influence intention to leave at Time 2, GEE was used to analyze whether all subscales of professional autonomy and OC each affected Time 2 intention to leave. Age, gender, education, and Time 1 intention to leave were used as control variables. Next, GEE was performed using the subscale of professional autonomy and OC that influenced Time 2 intention to leave, and the control variables.

The GEE approach, which is an extension of the generalized linear model, is well suited for repeated measures in longitudinal studies and for analyses with correlated structures, such as clustered data. The OC and professional autonomy treated in this study are presumed to have large variations in the difference in the amount of change among individuals. Therefore, the problem is overcome by inputting the variable changes (Time 2 − Time 1) in the longitudinal data at two points into the multiple regression model using the generalized estimation equation. In addition, eliminating the correlation effects of data from multiple hospital clusters was another reason for using GEE. Statistical processing was performed in SAS version 9.4 (SAS Institute Inc.: Cary, NC, USA). Statistical significance was set at *p* < 0.05 (two-sided).

## 3. Results

At Time 1, 11,171 nurses were approached, and 5768 nurses returned the questionnaire (response rate = 51.6%). At Time 2, 4100 nurses were approached—only those who provided their personal information in the first survey—and 2161 nurses returned the questionnaire (response rate = 52.7%). The data from 1778 nurses who were working in the same hospital at Time 1 and Time 2 and who responded to both questionnaires completely were analyzed (valid response rate = 15.9%). The mean age at Time 1 was 38.8 years (±10.2), and the mean number of years of nursing experience was 16.1 (±10.2). Of all participants, 1702 (95.7%) were women. Regarding educational background, 1527 (85.9%) went to vocational schools or junior colleges for registered nurses, and 251 (14.1%) attended a baccalaureate program (four-year program in nursing) or masters’ program in nursing. Table 1 shows the change in participant characteristics between Time 1 and Time 2.

Table 2 shows the descriptive statistics and results of the paired *t*-tests of each variable at Time 1 and Time 2. Concerning OC, the mean scores of the continuous OC at Time 2 were slightly higher (delta *M* = 0.43, *p* < 0.001), while those of normative OC were slightly lower (delta *M* = −0.27, *p* < 0.001). Concerning professional autonomy, the mean scores for all three subscales at Time 2 were higher (delta *M* = 0.54, 0.54, 0.49, for the three subscales, respectively; *p* < 0.001).

Regarding the correlation coefficient of the independent variables related to intention to leave at Time 1 and Time 2, intention to leave at Time 2 was significantly correlated with all subscales of professional autonomy and OC. Regarding rank stability, intention to leave was *r* = 0.53, autonomous clinical judgment *r* = 0.61, control over work conditions *r* = 0.62, job-related independence *r* = 0.69, affective OC *r* = 0.73, continuance OC *r* = 0.60, and normative OC *r* = 0.61, all significant at the *p* < 0.001 level. These results show that professional autonomy and OC were relatively stable within the individual, and affective OC was particularly highly stable. Although the use of years of nursing experience as a control variable was considered during the GEE analysis, the correlation coefficient between age and years of nursing experience was very high at *r* = 0.95 (*p* < 0.001). Therefore, it was not used because of the influence of multicollinearity.

**Figure 2:**
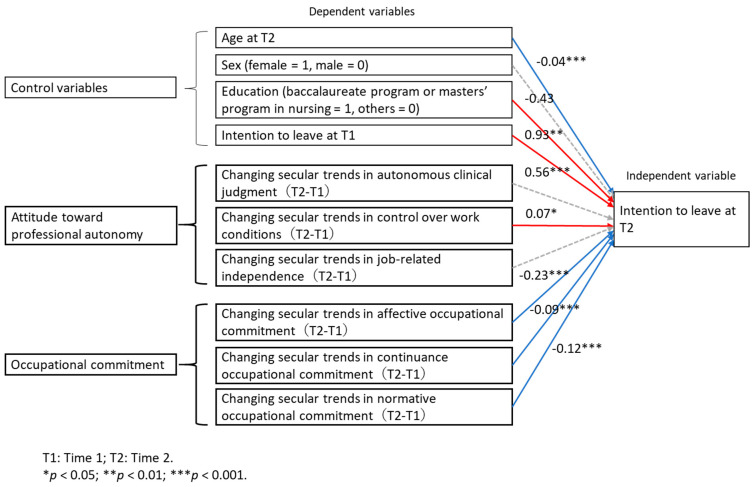
Generalized estimating equation results. The solid blue arrows represent that increasing age and increasing changing secular trends in affective OC, continuance OC, and normative OC decrease intention to leave at Time 2. The solid red arrows represent that higher education, increasing intention to leave at Time 1, increasing changing secular trends in control over work conditions increase intention to leave at Time 2. The gray dotted arrow indicates that there was no significant effect.

Table 3 shows the GEE results. First, using age, sex, education, and intention to leave at Time 1 as control variables, we analyzed whether all subscales of professional autonomy and OC respectively affect intention to leave at Time 2. Changing secular trends in the three subscales of OC and control over work conditions affected intention to leave at Time 2. Therefore, using control variables and these variables, the GEE revealed that education, intention to leave at Time 1, and changing secular trends in control over work conditions were confirmed as factors that had a significant positive influence on nurses’ intention to leave at Time 2. In other words, a higher level of education, increasing the intention to leave at Time 1, and increasing changes in secular trends in control over work conditions increased the intention to leave at Time 2. Conversely, age and changing secular trends in affective OC, continuance OC, and normative OC were confirmed as factors that had a significant negative influence on nurses’ intention to leave their jobs at Time 2. In other words, increasing age and increasing changing secular trends in affective OC, continuance OC, and normative OC decreased the intention to leave at Time 2. Sex and changing secular trends in autonomous clinical judgment and in job-related independence were found to have no effect on nurses’ intention to leave at Time 2. The effect on intention to leave at Time 2 is shown in Figure 2.

## 4. Discussion

We conducted a large-scale, longitudinal study targeting Japanese nurses with the aim of exploring the causal relationships between professional autonomy, OC, and nurses’ intention to leave. We clarified that increasing changes in secular trends in control over work conditions, which is a subscale of attitudes toward professional autonomy, increased nurses’ intention to leave at Time 2, while increasing changes in secular trends in all subscales of occupational commitment decreased their intention to leave at Time 2. Additionally, affective OC had a high mean stability at the population level, continuance OC had a significant mean increase in the population over two years, and normative OC decreased slightly. Moreover, nurses’ professional autonomy and OC were relatively stable in individuals, with affective OC being particularly stable. To our best knowledge, this is the first longitudinal study to examine the effects of the changes in nurses’ professional autonomy and OC on their intention to leave by adopting a measurement method that reflects the context of the countries in which nurses have limited autonomy. This is also the first study to reveal secular changes in groups and individuals in all attitudes toward professional autonomy and OC components. Therefore, our findings contribute to the current knowledge on nurses globally aiming to provide high-quality and safe medical care while also preserving their well-being.

Continuance OC exhibited a significant increase in population mean over approximately two years, with a slight decrease in normative OC in population mean. Six-month span measurements in nursing students have shown a reduction in both continuance and normative OC [33], but this study’s results supported this only partly. The difference between the results of the previous study and our study may be due to the fact that we did not include students, but nursing staff, and that the measurement span was longer. As seen in Table 1, the number of married people increased slightly over the two years, as did the number of nursing staff who took management positions. However, although a review of previous studies suggests that there is a relationship between job promotions and the level of affective OC, these studies are few, thus the relationship between the occurrence of life events, changes in job position, and normative/continuance OC is not clear [30]. Improved OC affects patient safety, perceived quality of care [31], and new nurses’ competence [4]. Therefore, it is necessary to further study what factors can increase nurses’ continuance and normative OC.

The present study revealed that the mean score of professional autonomy significantly increased across all three subscales. This is consistent with previous research, which indicated that nurses with many years of experience have higher professional autonomy [39]. However, a previous cross-sectional study measured behavioral autonomy in a country where nurses have discretionary power [39], and indicated that there was a limit to understanding the changes in the autonomy of nurses working in countries where their discretion is limited. The results of the current study provide new knowledge that, by adopting a measurement method that reflects the context of the country, the liberal attitude toward professional autonomy among nurses working in countries with limited autonomy improves over time. This suggests that nurses working in countries with limited discretion, such as Japan, Turkey, and Iran, are also improving their liberal attitude toward professional autonomy. This is important because it can be expected that professional autonomy will be exerted at the behavioral level if the nurses’ discretion over medical conduct is expanded.

The result that nurses’ OC was relatively stable is consistent with the findings of previous studies, which reported nurses’ OC over a six-month period [36]. In addition, the result that nurses’ attitudes toward professional autonomy was relatively stable is unique to this study. These results contribute to the existing literature by indicating that all subscales of nurses’ attitudes toward professional autonomy and OC are relatively stable in individuals, even over a two-year period. The within-individual stability of attitudes toward professional autonomy suggests that it gradually increases within an individual with significant increases in the mean population. As the current research population included nurses in various age groups, including newly graduated nurses, the personal stability of OC suggests that it may be stable during nurses’ early careers, as OC begins to form during the nursing course, in which students undergo vocational training [44].

Increasing the secular change in control over work conditions, which is a subscale of professional autonomy, has a significant influence on increasing nurses’ intention to leave; however, changing secular trends in autonomous clinical judgment and in job-related independence did not affect intention to leave in this study. Thus, results did not support hypothesis 1. This suggests that Japanese nurses whose progressive attitudes toward discretion of control over work conditions have increased over the course of their career may have enhanced their intention to leave. Since nurses who are dissatisfied with the flexibility of work style have a high intention to leave their jobs [11], nurses’ desire to manage working conditions themselves can lead to their moving to an organization with more flexible working conditions. Therefore, one way to reduce nurses’ intention to leave is giving them greater autonomy in managing their working conditions.

In addition, the result that secular changes in autonomous clinical judgment and in job-related independence did not affect nurses’ intention to leave supported the result of a survey that targeted registered nurses in the Philippines [3]. However, there are multiple factors that influence nurses’ professional autonomy, and these factors and their level of impact vary from country to country [24]. Thus, one must be careful in interpreting these results. Appropriate measures of the relationship between nurses’ professional autonomy and turnover intentions need to be taken in different cultures, and the results should be integrated.

We clarified that increasing changing secular trends in affective, continuance, and normative OC have a significant influence on decreasing nurses’ intention to leave, thus supporting hypothesis 2. Previous cross-sectional studies reported that increasing OC decreased nurses’ intention to leave [16,17,18]. In this study, we provided new knowledge by considering secular changes in OC. This finding was expected, and suggests that as OC increases over time, intention to leave decreases. Lee et al. [29] stated that a person with strong OC will identify more strongly with and experience more positive feelings about the occupation than one with a weak OC; thus, it is presumed that as OC increases over the course of a nurse’s career, intention to leave will decrease. Therefore, it is necessary to continually improve nurses’ OC to decrease turnover.

The results also show that increasing changing secular trends in all three components of OC decrease the intention to leave. It has been clarified that nurses’ OC is affected by educational history, which also influences intention to leave [45]. Thus, it is possible to maintain and improve nurses’ OC by continually implementing educational programs. However, the influence on each specific component of OC—affective, continuance, and normative—has not been verified; therefore, further research is needed to guide the development of specific educational programs to improve nurses’ long-term professional commitment and reduce their intention to leave.

## 5. Study Limitations

This study had some limitations. We targeted nurses solely from a hospital group in western Japan, which may limit generalizability. Therefore, further research should broaden the geographical scope and search for factors influencing secular trends in attitudes toward professional autonomy and OC, such as age, years of nursing experience, nurse type, and hospital characteristics. Clarifying the factors that affect attitude changes can lead to the development of educational programs for nurses that may reduce their intention to leave their jobs.

Furthermore, our study showed that affective OC has high individual stability and high mean stability at the population level. Previous research analyzed whether exposure to threatening physical and verbal events in the workplace affected nurses’ affective OC, and reported that experiencing verbal abuse and being injured in the workplace was detrimental to affective OC [46]. In this study, we reported high stability of affective OC over about two years, but negative physical and mental events may cause a temporary increase/decrease in nurses’ affective OC. Our study did not focus on providing data on these work settings, including negative work settings such as threatening physical events, positive work settings such as boss support, and general work settings such as team size; however, such aspects are important and must be investigated in future studies.

## 6. Conclusions

This study found that nurses’ professional autonomy and OC were relatively stable in individuals over approximately two years. In addition, affective OC was highly stable at the population level, continuance OC showed a significant increase, and normative OC decreased slightly. It can be suggested that nurses working in countries with limited autonomy, such as Japan, can improve their liberal attitudes toward professional autonomy, as group averages increased for all three subscales of attitudes toward professional autonomy. Moreover, increasing secular changes in nurses’ control over work conditions had a significant positive impact on increasing their intention to leave, and increasing secular changes in affective, continuance, and normative OC had a significant negative impact on decreasing nurses’ intention to leave. Further research is necessary to investigate factors that change professional autonomy and OCs over time in order to reduce nurses’ turnover behavior and to provide high-quality, safe medical care.

### Implications for Practice

Our findings suggest that nurses with a progressive attitude toward discretion of control over work conditions may wish to change jobs; therefore, increasing nurses’ autonomy over work conditions (e.g., introduction of a full-time working hours selection system, flexible selection of length and frequency of night shifts, flexible selection method of how to take vacation days, etc.) should reduce their intention to leave. Additionally, it is necessary to enhance nurses’ affective, continuance, and normative OC from the nursing student stage over time by implementing educational programs that promote professional development for reducing turnover and providing safe medical care.

## Figures and Tables

**Table 1:** Change in participants’ basic attributes between Time 1 and Time 2 (*n* = 1778).

Variable	Time 1, *n* (%)	Time 2, *n* (%)
Marital status		
Married	980 (55.1)	1049 (59.0)
Single	778 (43.8)	714 (40.2)
Employment conditions		
Full-time employees	1671 (94.0)	1683 (94.7)
Part-time employees	102 (5.7)	95 (5.3)
Position		
Director	7 (0.4)	11 (0.6)
Deputy director	16 (0.9)	17 (1.0)
Head nurse	145 (8.2)	162 (9.1)
Assistant head nurse	191 (10.7)	214 (12.0)
Regular nurse	1411 (79.4)	1368 (76.9)

**Table 2:** Descriptive statistics of nurse variables at Time 1, Time 2, and Time 2 − Time 1 (*n* = 1778).

Variable	Time 1		Time 2		Time2 - Time1		*t*-Test ^‡^
	Mean	SD ^†^	Mean	SD ^†^	Mean	SD ^†^	
Intention to leave	13.00	4.8	12.98	4.9	−0.02	4.7	ns
Occupational commitment							
Affective occupational commitment	22.00	3.9	22.08	4.0	0.08	2.9	ns
Continuance occupational commitment	17.49	3.8	17.92	3.8	0.43	3.4	***
Normative occupational commitment	17.63	3.6	17.36	3.8	−0.27	3.3	***
Attitude toward professional autonomy							
Autonomous clinical judgment	25.80	3.7	26.34	3.6	0.54	3.2	***
Control over work conditions	18.90	4.3	19.44	4.3	0.54	3.7	***
Job-related independence	15.60	4.1	16.09	4.0	0.49	3.2	***

† standard deviation; ‡ Result of a paired *t*-test between Time 1 score and Time 2 score; ***p < 0.001; ns = not significant.

**Table 3:** Generalized estimating equation results (*n* = 1778).

Parameter	Bivariate			Multivariate		
	β		SE^ †^	β		SE^ †^
Intercept				0.74		0.38
Age at Time 2				−0.04	***	0.01
Sex (female = 1, male = 0)				−0.43		0.31
Education (baccalaureate program or masters’ program in nursing = 1, others = 0)				0.93	**	0.34
Intention to leave at Time 1				0.56	***	0.02
Changing secular trends in autonomous clinical judgment	−0.05		0.03			
Changing secular trends in control over work conditions	0.07	*	0.03	0.07	*	0.03
Changing secular trends in job-related independence	0.00		0.04			
Changing secular trends in affective occupational commitment	−0.27	***	0.03	−0.23	***	0.03
Changing secular trends in continuance occupational commitment	−0.14	***	0.03	−0.09	***	0.02
Changing secular trends in normative occupational commitment	−0.18	***	0.02	−0.12	***	0.02

^†^ standard error; * *p* < 0.05; ** *p* < 0.01; *** *p* < 0.001.
